# The association between insurance status and in-hospital mortality on the public medical wards of a Kenyan referral hospital

**DOI:** 10.3402/gha.v7.23137

**Published:** 2014-02-11

**Authors:** Geren S. Stone, Titus Tarus, Mainard Shikanga, Benson Biwott, Thomas Ngetich, Thomas Andale, Betsy Cheriro, Wilson Aruasa

**Affiliations:** 1 Department of Medicine, Center for Global Health, Massachusetts General Hospital, Boston, MA, USA; 2 Department of Medicine, Harvard Medical School, Boston, MA, USA; 3 Department of Medicine, Indiana University School of Medicine, Indianapolis, IN, USA; 4 Department of Medicine, Moi University, Eldoret, Kenya; 5 Moi Teaching and Referral Hospital, Eldoret, Kenya

**Keywords:** health insurance, disparities, hospital medicine, Africa

## Abstract

**Background:**

Observational data in the United States suggests that those without health insurance have a higher mortality and worse health outcomes. A linkage between insurance coverage and outcomes in hospitalized patients has yet to be demonstrated in resource-poor settings.

**Methods:**

To determine whether uninsured patients admitted to the public medical wards at a Kenyan referral hospital have any difference in in-hospital mortality rates compared to patients with insurance, we performed a retrospective observational study of all inpatients discharged from the public medical wards at Moi Teaching and Referral Hospital in Eldoret, Kenya, over a 3-month study period from October through December 2012. The primary outcome of interest was in-hospital death, and the primary explanatory variable of interest was health insurance status.

**Results:**

During the study period, 201 (21.3%) of 956 patients discharged had insurance. The National Hospital Insurance Fund was the only insurance scheme noted. Overall, 211 patients (22.1%) died. The proportion who died was greater among the uninsured compared to the insured (24.7% vs. 11.4%, Chi-square=15.6, *p*<0.001). This equates to an absolute risk reduction of 13.3% (95% CI 7.9–18.7%) and a relative risk reduction of 53.8% (95% CI 30.8–69.2%) of in-hospital mortality with insurance. After adjusting for comorbid illness, employment status, age, HIV status, and gender, the association between insurance status and mortality remained statistically significant (adjusted odds ratio (AOR)=0.40, 95% CI 0.24–0.66) and similar in magnitude to the association between HIV status and mortality (AOR=2.45, 95% CI 1.56–3.86).

**Conclusions:**

Among adult patients hospitalized in a public referral hospital in Kenya, insurance coverage was associated with decreased in-hospital mortality. This association was comparable to the relationship between HIV and mortality. Extension of insurance coverage may yield substantial benefits for population health.

In 2005, the 58th World Health Assembly called for health systems to move toward universal coverage and social health insurance in order ‘to guarantee access to necessary services while providing protection against financial risk’ (1). Hailed as one of the great achievements in financing healthcare in the past century, insurance-based financing represented a move away from direct out-of-pocket payments. Today most industrial countries provide universal access to healthcare through a combination of social insurance, private insurance, general revenues, and user charges (2). Risk-sharing mechanisms such as social insurance provide resources to access healthcare and to promote health while protecting individuals and households against the potentially devastating direct financial costs of illness.

In Kenya, the National Hospital Insurance Fund (NHIF) is the primary provider of health insurance with a mandate to enable all Kenyans to access quality and affordable healthcare services (3–5). NHIF has existed since its establishment by Parliament in 1966 shortly after Kenya achieved its independence in 1963. Initially part of the Ministry of Health, NHIF was restructured by the enactment of the NHIF Act of 1998. Membership is compulsory for all salaried workers in the formal sector (both public and private) at a monthly cost of 30–320 KSH (US$0.34–3.65) depending on salary. Membership is also available on a voluntary basis to informal sector workers at a cost of 160 KSH (US$1.83) per month. The benefits package includes comprehensive inpatient medical coverage including fees of up to 396,000 KSH (US$4,521) per year for the contributors as well their dependents. The type of healthcare facility determines co-payments and cost sharing. Patients have no co-payment obligations when receiving inpatient care at government facilities, but are often responsible for some co-payments at private hospitals. By 2011, approximately 2.7 million Kenyans were members of the NHIF including 2.1 million formal sector employees. With the insurance benefits extending to approximately another 6 million dependents, including spouses, children, and disabled family members, it is estimated that insurance coverage had increased to approximately 20% of Kenya's population receiving insurance coverage, with only a small percentage obtaining coverage through private, community-based, or employer-based programs (5, 6).

While observational data from past decades in the United States suggest that those without insurance generally have higher mortality and worse health outcomes (7–17), there is no published literature examining health outcomes associated with insurance coverage in this context. Past work in East Africa examining Rwanda's community-based insurance program *Mutuelles de santé* has demonstrated improved medical care utilization and financial risk protection among insured household (18, 19) Similarly, findings have been demonstrated in the West African nations of Burkina Faso, Ghana, Senegal, and Mali (20, 21). It is hoped that improved access and utilization will promote improved health outcomes, and the growth of insurance coverage in Rwanda has coincided with significant decreases in under-five mortality, infant mortality, and maternal mortality (19). Yet, unlike in the United States, the relationship between insurance coverage and health outcomes in hospitalized patients has yet to be firmly demonstrated in resource-poor settings such as Kenya.

The objective of this study was to determine whether uninsured patients admitted to the public medical wards at a Kenyan referral hospital have any difference in in-hospital mortality rates from those patients with insurance.

## Methods

### Study design and setting

This study was a retrospective observational study of patients admitted to the public medical wards at Moi Teaching and Referral Hospital (MTRH) for the 3-month period from October to December 2012. Located in city of Eldoret, MTRH is an approximately 750-bed national referral hospital for western Kenya. The public medical wards admit and discharge approximately 350 patients monthly. Those in the lowest socioeconomic strata largely populate these wards as the wealthier largely choose private wards or hospitals (22). Eldoret is located in Rift Valley Province, where the health insurance coverage was 11.9% in 2007, primarily due to coverage by NHIF (6). Across Kenya, approximately 45.9% of the population lives below the poverty line with an unemployment rate of approximately 40% (23, 24). The study was approved by the Institutional Research Ethics Committee at Moi University College of Health Sciences and was submitted and determined to be exempt from review by the institutional review board at Indiana University.

### Population

All patients discharged from the public medical wards at MTRH between October 1, 2012, and December 31, 2012, were eligible for inclusion in the study.

### Data collection

Data were retrieved from MTRH's medical record database. Following each patient's death or departure from the hospital, the Medical Records Department examines the patient's paper file recording demographic and clinical information. The primary outcome of interest was in-hospital death. The primary explanatory variable of interest was health insurance status. In addition to these variables, we also abstracted information on primary and secondary diagnoses (ICD-10 codes), HIV status, age, gender, occupation, and length of hospitalization as determined from admission to medical discharge.

All patient identifiers were absent from abstracted data utilized for analysis for this study.

### Statistical analysis

The bivariate association between insurance status and mortality was examined using a Chi-square test. We then fit a logistic regression model to the data with in-hospital mortality as the dependent variable and insurance status as the primary explanatory variable. We adjusted this estimate for age, gender, employment status, presence of a secondary comorbid illness, and HIV status (as gathered from primary or secondary diagnostic codes) in order to calculate an adjusted odds ratio (AOR).

All analyses were conducted with IBM SPSS Statistics software (Version 21; IBM Corp. Armonk, New York).

## Results

### Study population

Over the 3-month period from October 1, 2012, to December 31, 2012, a total of 956 patients were discharged from the public medical wards at MTRH. The average age of patients discharged during this time was 41.8 years old (range 13–102, SD 18.9). There were 519 men (54.3%) and 437 women (45.7%) in the population. Additionally, 62% of this population was employed outside the household, and 10.6% of patients had been referred from an outside facility. This population represented 324 different ICD-10 diagnoses with the leading primary diagnoses including HIV-associated illnesses [189 (19.8%)], poisonings [87 (9.1%)], congestive heart failure [74 (7.7%)], neurologic conditions (i.e. stroke, neuropathy, paralysis, seizures) [66 (6.9%)], psychiatric conditions (i.e. psychosis, depression) [56 (5.9%)], diabetes mellitus [52 (5.4%)], pneumonia [47 (4.9%)], anemia [46 (4.6%)], hypertension [43 (4.5%)], tuberculosis [38 (4.0%)], and gastroenteritis [32 (3.3%)]. Approximately 20.4% of the patients had comorbid illnesses in addition to their primary indications for admission. The most frequent secondary diagnosis was HIV, indicated for 122 of 195 (62.6%) cases with a secondary diagnosis. Overall, 20.8% of the discharged patients had HIV included as either a primary or secondary diagnosis for their hospitalization. The average length of stay was 8.6 days (range 1–100, SD 9.4). Overall, 21.3% of discharged patients had insurance through NHIF. There were no patients from the medical wards that were recorded as having private or other insurance coverage.

Overall, 21.3% of discharged patients had insurance through NHIF. There were no patients from the medical wards that were recorded as having private or other insurance coverage. Those patients with NHIF were significantly more likely to be employed outside the household and to have a comorbid illness secondary to the primary reason for hospitalization (Table 1). Otherwise, the populations did not differ significantly with respect to age, gender distribution, referral status, length of stay, and HIV prevalence.

**Table 1:** Study population

	Uninsured	Insured	Difference
N	744	201	–
Age	42.2 yr	39.9 yr	+2.3 (*p*=0.08)
% Male	*(range 14–100, SD 19.6)*	*(range 13–82, SD 15.3)*	
% Male	53.10%	57.80%	−4.7% (*p*=0.28)
Employed*	60.00%	70.20%	−10.2% (*p*=0.01)
Referred†	10.30%	10.40%	−0.1% (*p*=1.0)
No. of primary diagnoses	1.36	1.38	−0.02 (*p*=0.67)
% HIV	21.10%	20.40%	−0.7% (*p*=0.92)
% Comorbid illness‡	18.70%	27.40%	−8.7% (*p*<0.01)
Length of stay	8.63 d *(range 1–100, SD 9.9)*	8.52 d *(range 1–49, SD 7.7)*	+0.11 (*p*=0.88)

* Employment status categorized in binary fashion—employed outside home or not

† % of patients referred from outside facility

‡ % of patients with secondary diagnosis recorded.

### Outcome

**Fig. 1:**
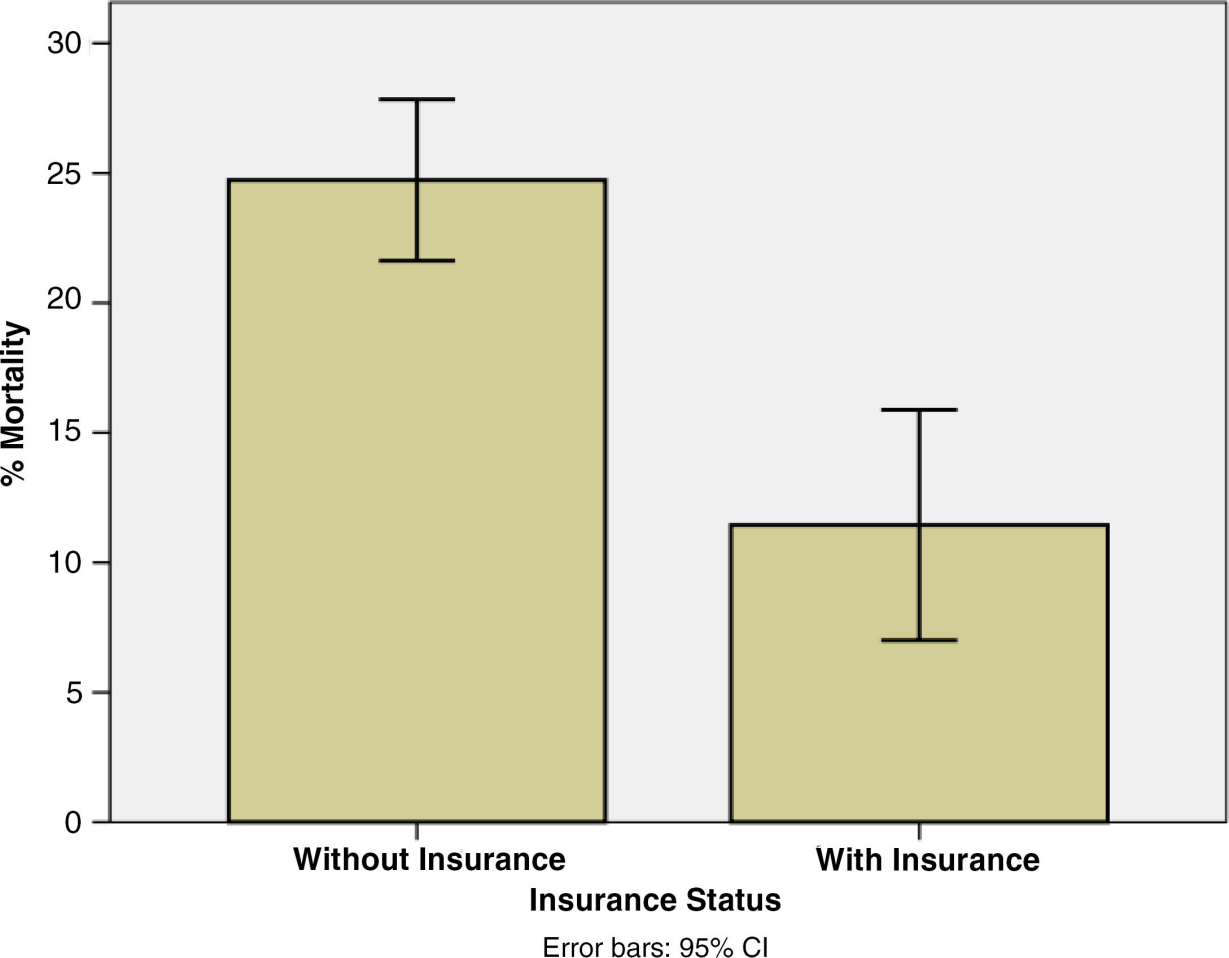
Insurance status and mortality. The mortality percentage for patients categorized by whether they had insurance (*p*<0.001).

The overall mortality rate of patients discharged from the medical wards during the study period was 22.1% (95% CI 19.5–24.7%). The mortality rate for uninsured patients was 24.7% while the rate for insured patients was 11.4% (Fig. 1). This equates to an absolute risk reduction of 13.3% (95% CI 7.9–18.7%) and a relative risk reduction of 53.8% (95% CI 30.8–69.2%) for mortality. Moreover, a Chi-square test for independence demonstrated a Chi-square value of 15.6 (*p*<0.001) supporting a significant bivariate association between insurance status and mortality in this population.

In standard multiple logistic regression examining the association between insurance status, age, gender, employment, presence of a secondary comorbid illness, HIV status, and mortality, the model containing all predictors was statistically significant (*p*<0.001). As shown in Table 2, mortality was independently associated with age (AOR 1.02, 95% CI 1.01–1.03), HIV status (AOR 2.45, 95% 1.56–3.86), and insurance status (AOR 0.40, 95% CI 0.24–0.66).

**Table 2:** Multiple logistic regression predicting mortality

	B	*p*	Odd ratio	95% CI
Age	0.016	<0.001	1.02	1.01–1.03
Gender	0.043	0.83	1.04	0.72–1.54
Insurance status	−0.917	<0.001	0.40	0.24–0.66
Employment status	0.387	0.06	1.47	0.98–2.22
Comorbid illness	−0.304	0.23	0.74	0.45–1.21
HIV status	0.895	<0.001	2.45	1.55–3.86

Results of standardized logistic regression with the dependent variable of patient mortality.

## Discussion

Analyzing data from 956 patients discharged over a 3-month period at a Kenyan referral hospital, our study demonstrates that individuals with health insurance had significantly lower all-cause in-hospital mortality as compared with patients without insurance. The in-hospital mortality rate for insured patients was approximately 54% lower than uninsured patients. This remained statistically significant even after controlling for age, gender, employment, presence of a secondary comorbid illness, and HIV status. Moreover, after controlling for these confounders, insurance coverage had an association of comparable magnitude to that of HIV with in-hospital mortality for adult medical inpatients. The significant difference in mortality between insured and uninsured inpatients may reflect unmeasured differences in the populations as well as differences in healthcare provided for hospitalized patients.

In this population, insured patients were more likely to have a comorbid secondary or chronic illness. These diagnoses largely represent chronic medical problems such as hypertension or diabetes. These problems could be more represented in the insured population as this population is better able to access care for diagnosis and management than the uninsured. Yet, as the NHIF insurance scheme only covers inpatient hospitalizations currently, any difference in outpatient care provision for such conditions would be due more to differences in socioeconomic status, education, and health seeking behaviors rather than insurance coverage itself.

Furthermore, past work has demonstrated the rate of hospital admission among Kenyans with insurance was approximately 48 admissions per 1,000 per year, compared to 28 admissions per 1,000 per year among the uninsured (6). While representing a small fraction of the entire population, this increased admission rate amongst the insured may reflect increased access and earlier care-seeking behavior. Insured patients, who may be more educated and live in urban areas, are able to recognize appropriate conditions warranting healthcare and access the care needed. The uninsured alternatively may delay accessing healthcare due to barriers in cost and geography as well as a possible lack of recognition of need. Ministry of Health data from 2007 demonstrated that lack of money along with self-medicating were the most common reasons for ill patients not seeking care (6). Additionally, another study suggested that the richest individuals in Kenya are nearly three times more likely than the poorest to access healthcare when needed (25). Moreover, the poorest not only utilized fewer services when needed but also reported more frequently being unable to complete the whole course of treatment recommended (25).

Beyond pre-existing differences prior to admission, the differences in mortality between insured and uninsured inpatients may also be due to differences in care once admitted. At MTRH, payments are required of patients prior to receiving certain diagnostic tests, including echocardiograms, x-rays, and CT scans. Thus, there can be delays in vital diagnostic tests and management decisions as patients and their families seek funds. There are waiver mechanisms for patients unable to pay and for emergently needed tests, but even the waiver process can take days. Patients with insurance, on the other hand, are able to get these tests more expediently as the hospital will perform the test with insurance reimbursement upon discharge. Overall, healthcare providers are able to diagnose and treat patients with insurance more expeditiously without as many barriers to care. This difference in care provision may impact the ultimate outcomes of the patients and partially explain some of the differences in mortality.

Our study had a number of limitations that need to be highlighted. First, the dataset did not contain information with respect to socioeconomic status and education level for which insurance status may be acting as a surrogate marker. Moreover, our dataset did not allow for classification of residence (urban versus rural) or a means of calculating distance from residence to hospital in order to identify if any of these factors may have contributed to the differences seen. Generally, past studies in Kenya have shown that patients with insurance are richer, more educated, more likely to be employed, and more likely to have an urban residence (6, 25–27). All of these factors may contribute to increased access to care and improved outcomes; however, beyond employment status, our dataset did not provide other measures of patients’ socioeconomic status, education level, or place of residence for comparison. Also, the data collected did not include markers of the severity of illness upon presentation or capture any delays in care provision either before or during the hospitalization that may have been due to lack of money and insurance.

## Conclusion

Risk-sharing mechanisms such as social insurance provide resources to access healthcare and to promote health while protecting individuals and households against these potentially devastating direct financial costs of illness. While observational data from past decades in the United States suggest that those without insurance generally have higher mortality and worse health outcomes (7–17), our study demonstrates that a similar association may be seen in adult medical inpatients at a public Kenyan referral hospital. Insurance coverage correlated with a 53.8% relative risk reduction in in-hospital mortality. This association remained significant after controlling for several confounders and was even comparable in magnitude to the relationship between HIV and in-hospital mortality. Our study is unable to answer if it is the insurance coverage itself or underlying differences in the populations of insured and uninsured patients that are responsible for this association. However, in a country with an annual per capita health expenditure of approximately US$37 (28), our study suggests that expanding inpatient health insurance coverage at a similar or even lower annual cost for an entire household could potentially have a substantial impact on patient health outcomes. Future research will need to examine what aspects of having insurance coverage account for this significantly lower in-hospital mortality rate in this setting. As Kenya moves toward its Vision 2030 and goal of ‘good health and reliable, equitable, affordable and sustainable healthcare services for the entire population of Kenya’ (28), these answers will be needed to direct efforts in the most meaningful and effective directions of which social insurance may prove to be ultimately one of the most valuable.
